# Update on Poly-ADP-ribose polymerase inhibition for ovarian cancer treatment

**DOI:** 10.1186/s12967-016-1027-1

**Published:** 2016-09-15

**Authors:** Anselmo Papa, Davide Caruso, Martina Strudel, Silverio Tomao, Federica Tomao

**Affiliations:** 1 Oncology Unit, Department of Medico-Surgical Sciences and Biotechnologies, University of Rome “Sapienza”, Latina, Italy; 2 Oncology Unit, Department of Radiological Sciences, Oncology and Pathology, University of Rome “Sapienza”, Latina, Italy; 3 Department of Gynaecology and Obstetrics, University of Rome “Sapienza”, Policlinico “Umberto I”, Rome, Italy

**Keywords:** Epithelial ovarian cancer, Target therapy, Poly-ADP-ribose polymerase inhibitor (PARPi), Breast related cancer antigens (BRCA)

## Abstract

**Background:**

Despite standard treatment for epithelial ovarian cancer (EOC), that involves cytoreductive surgery followed by platinum-based chemotherapy, and initial high response rates to these, up to 80 % of patients experience relapses with a median progression-free survival of 12–18 months. There remains an urgent need for novel targeted therapies to improve clinical outcomes in ovarian cancer. Of the many targeted therapies currently under evaluation, the most promising strategies developed thus far are antiangiogenic agents and Poly(ADP-ribose) polymerase (PARP) inhibitors. Particularly, PARP inhibitors are active in cells that have impaired repair of DNA by the homologous recombination (HR) pathway. Cells with mutated breast related cancer antigens (BRCA) function have HR deficiency, which is also present in a significant proportion of non-BRCA-mutated ovarian cancer (“BRCAness” ovarian cancer). The prevalence of germline BRCA mutations in EOC has historically been estimated to be around 10–15 %. However, recent reports suggest that this may be a gross underestimate, especially in women with high-grade serous ovarian cancer (HGSOC).

**Main body of the abstract:**

The emergence of the DNA repair pathway as a rational target in various cancers led to the development of the PARP inhibitors. The concept of tumor-selective synthetic lethality heralded the beginning of an eventful decade, culminating in the approval by regulatory authorities both in Europe as a maintenance therapy and in the United States treatment for advanced recurrent disease of the first oral PARP inhibitor, olaparib, for the treatment of BRCA-mutated ovarian cancer patients. Other PARP inhibitors are clearly effective in this disease and, within the next years, the results of ongoing randomized trials will clarify their respective roles.

**Conclusion:**

This review will discuss the different PARP inhibitors in development and the potential use of this class of agents in the future. Moreover, combination strategies involving PARP inhibitors are likely to receive increasing attention. The utility of PARP inhibitors combined with cytotoxic chemotherapy is of doubtful value, because of enhanced toxicity of this combination; while, more promising strategies include the combination with antiangiogenic agents, or with inhibitors of the P13K/AKT pathway and new generation of immunotherapy.

## Background

Epithelial ovarian cancer (EOC) accounts for 90 % of all ovarian cancers (OC) and typically presents in post-menopausal women [1].

In terms of incidence, it is the second most common malignant gynecological disease, the sixth most common cancer and the seventh most common cause of cancer death in women. Particularly, EOC is the most common cause of death from gynecological malignancy [2, 3].

Standard treatments for EOC involves cytoreductive surgery followed by platinum-based chemotherapy. For high-grade serous ovarian cancer (HGSOC), the most prevalent and aggressive form of EOC, relapse is nearly the norm because of the development of resistance, although approximately 80 % of patients initially respond to treatment [4, 5].

According to the response to systemic therapy, EOC can be divided in: platinum-refractory, if there is a progression within 1 month or stable disease (SD) during first-line therapy; platinum-resistant, if there is a response during therapy and a relapse within 6 months. Nevertheless recurrent EOC may be chemo-sensitive to platinum and therefore patients can be still treated with platinum-based chemotherapy; these are patients platinum-sensitive, if there is a relapse after 12 months from the therapy. Moreover, EOC patients relapsing between 6 and 12 months show an intermediate sensitivity to platinum; for this reason, they are called platinum-partially sensitive. Longer the interval from the end of platinum-based chemotherapy, better is the outcome using platinum again [6, 7].

Based on the classical abdominal spread, besides classical chemotherapy which has been in use over the years, an intraperitoneal chemotherapy has been developed [8].

The response rate (RR) to chemotherapy is high, with higher rate of relapse.

Therefore, recurrent EOC constitutes a poor prognosis disease, forcing patients to receive multiple lines of chemotherapy, with unsatisfactory results due to the occurrence of drug-resistant cancer clones. For these reasons, and in order to identify more appropriate therapeutic options, a considerable scientific interest towards the identification of alternative molecular pathways, involved in ovarian carcinogenesis and new drugs related, has developed [9].

Among these new compounds, anti-angiogenetic target therapies have shown activity in association with standard chemotherapy in several trials; particularly bevacizumab (BV) in four phase III trial showed an increase of progression free survival (PFS) and overall survival (OS) [10–12].

Although standard treatments can effectively reduce tumor mass, a lot of patients with residual ovarian cancer stem cells (CSCs), could acquire chemo-resistance [13–15].

In this context, the CSC theory supports that even if a small number of CSCs remains in situ after therapy, disease recurrence can occur [16]. Several CSC’ pathways could be involved in these mechanisms including activation of anti-apoptotic factors, inactivation of pro-apoptotic effectors, and/or reinforcement of survival signals [17–19].

With the advent of next-generation sequencing in recent years, EOC has been found to consist of a complex set of diseases. Diverse genetic or epigenetic alterations that are of fundamental importance in tumorigenesis and progression have been identified in heterogeneous subsets of patients [20].

For example, BRCA1/2 germ-line mutation is essentially associated with HGSOC [21]. The prevalence of germline breast related cancer antigens (BRCA) mutations (gBRCAm) in EOC has historically been estimated to be around 10–15 % [22]. Recent reports suggest that this may be a gross underestimate, especially in women with HGSOC [23].

The emergence of the DNA repair pathway as a rational target in various cancers led to the development of the PARP inhibitors (PARPi) [24].

PARPi exploit the mechanism of ‘synthetic lethality’, that is loss of function in two genes causes cell death as opposed to a non-lethal effect of functional loss of one of the genes, to target tumours with defective DNA repair mechanisms, such as aberrant homologous recombination (HR) repair due to loss of BRCA1 and 2 gene function through the presence of mutations [25].

Such data support the use of BRCA mutation testing in all patients with HGSOC, regardless of family history. This expansion in BRCA testing will require changes to the traditional genetic service pathways in which patients are screened and referred based on family history, moving to a more streamlined oncology-based genetic testing service.

Determining the molecular events that control this tumour trait might advance the understanding of tumorigenesis and facilitate individualized treatment strategies for this lethal disease.

## BRCA and PARP role in DNA stability system

DNA continually sustains damaging alterations under a constant barrage of environmental insults, toxic products of metabolism, and erroneous DNA replication. These alterations can be divided into: base modifications; single strand breaks (SSB); double strand breaks (DSB); and intrastrand or interstrand cross-links [26].

**Fig. 1:**
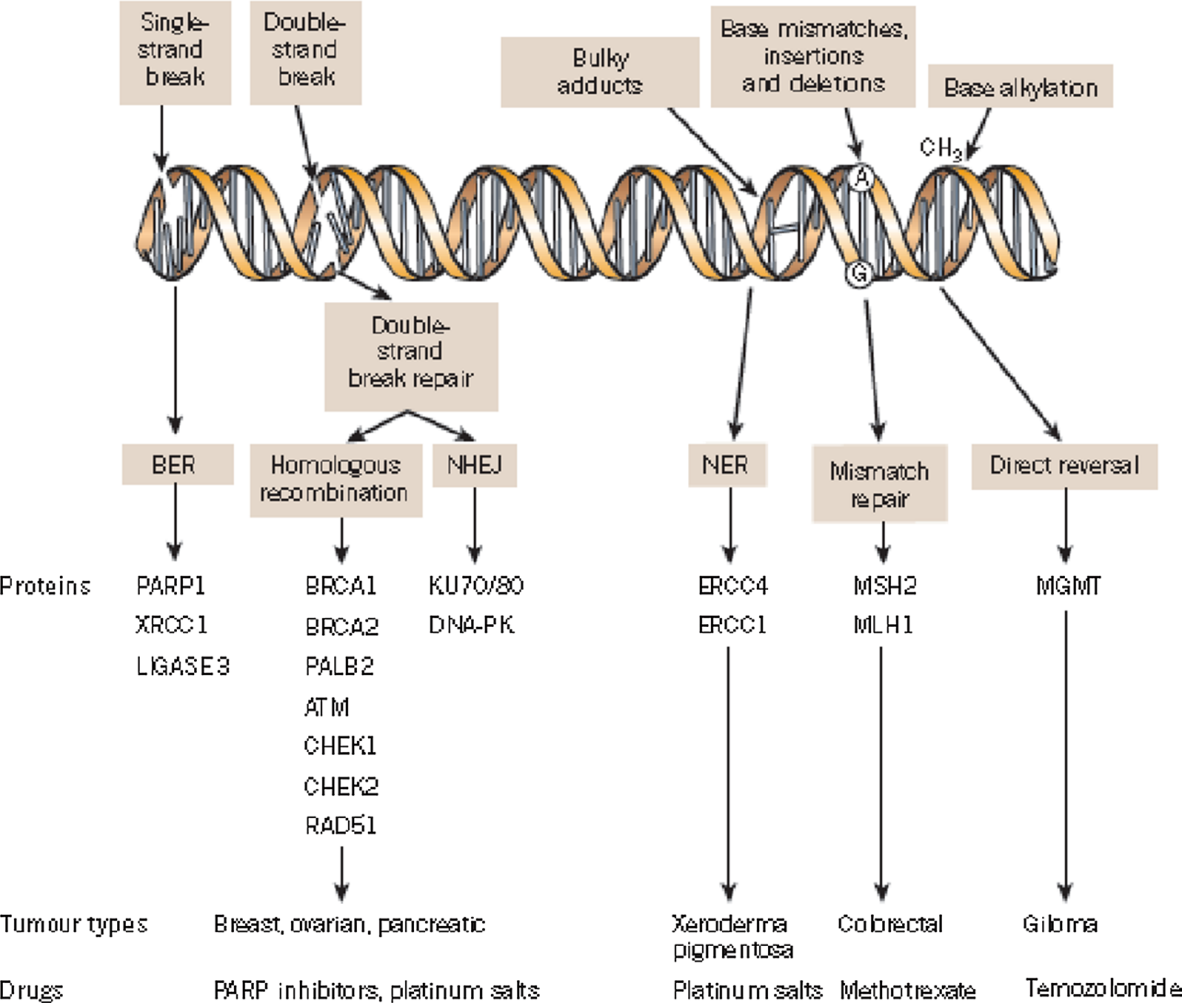
A panoply of DNA repair mechanisms maintains genomic stability. DNA is continually exposed to a series of insults that cause a range of lesions, from single-strand breaks (SSBs) to base alkylation events. The choice of repair mechanism is largely defined by the type of lesion, but factors such as the stage of the cell cycle also have a role. Key proteins involved in each DDR mechanism, the tumour types usually characterized by DDR defects and the drugs that target these defects are shown. *BER* base excision repair, *NER* nucleotide excision repair, *NHEJ* non-homologous end-joining. Figure modified, with permission, from Lord et al. [24]

Several DNA repair mechanisms have evolved to repair these lesions and maintain genomic integrity. Given the large array of potential lesions and the importance of high-fidelity repair, DNA repair mechanisms are generally complex, highly redundant, and to a large extent conserved across phylogenetic classes [27] (Fig. 1).

SSB repair mechanisms include base excision repair (BER), nucleotide excision repair (NER) and mismatch repair (MMR) pathways [28].

Amongst the various DNA insults, single strand alterations occur most often at a rate of approximately 104 per day and are repaired through a combination of BER, NER and MMR mechanisms using the intact DNA strand as a template. The predominant pathway of SSB repair is the BER utilizing PARP [29].

PARP is a nuclear protein that senses and binds to DNA SSB and subsequently activates the BER pathway by recruiting additional repair factors. Of the 17 known members of the PARP super-family in humans, PARP-1 accounts for more than 90 % cellular DNA repair activity and remains the most studied [30, 31].

PARP-1 is recruited and activated by SSBs as a homodimer in a fast reaction and upon binding to a damaged strand; PARP-1 undergoes a conformational change inducing the C-terminal catalytic domain to transfer ADP-ribose moieties from cellular nicotinamide-adenine-dinucleotide (NAD+) to protein acceptors, including the central auto-modification domain of PARP1 itself. The major mechanism that limits the PAR-ylation of protein acceptors is PAR hydrolysis by Poly-(ADP-ribose) glucohydrolase (PARG). The amount of PAR present in the cell depends on the balance between PARP1, and PARG. PARP-1 function is restored by the degradation of PAR. In case of small to moderate damage, PARP-1 allows for the restoration of genomic integrity and the return to normal cellular function. However, emerging evidence has implicated PARP-1 over activation in unregulated PAR synthesis, depleting NAD, and consequently ATP, eventually leading to widespread cell death. In this recently characterized model, PARP-1 over activation results in the synthesis of numerous long branched PAR polymers which triggers the translocation of apoptosis-inducing factor from mitochondria to the nucleus resulting in caspase-independent cell death [32].

The lengthening PAR chain builds up a large negatively charged structure at the SSB which recruits other DNA repairing enzymes. These include DNA ligase III (LigIII), DNA polymerase beta (polβ), and scaffolding proteins such as X-ray cross complementing gene 1 (XRCC1), that collectively form the BER multi-protein complex. Among the proteins it recruits, XRCC1 is crucial for DNA repair, initially assembling and activating the BER machinery through the modification of several proteins such as histones and topoisomerases but subsequently “switching off” the BER machinery by decreasing the affinity of both histones and PARP-1 to DNA. As it dissociates from DNA, PARP-1 becomes inactive and no further synthesis of the PAR polymer occurs [33].

Regarding DSB repair mechanisms, they consist of HR and non-homologous end-joining (NHEJ) pathways. HR consists of gene conversion and single-strand annealing pathways. This pathway is mostly active in the S and G2 phases of the cell cycle. Crucial proteins involved in mediating homologous recombination include those encoded by the BRCA1, BRCA2, RAD51 and PALB2 genes. The BRCA1 and BRCA2 proteins are important in maintaining genomic stability by promoting efficient and precise repair of DSB [34, 35] (Fig. 1).

## PARP inhibitors in BRCA mutated EOC and synthetic lethality concept

PARPi mediate their anti-cancer effects as catalytic inhibitors blocking repair of DNA SSBs by the BER/SSBR pathway (Fig. 1). The initial clinical development of PARPi focused on their role as chemo-sensitizers, and their single-agent activity was unknown. Ten years ago two articles published in Nature reported that BRCA1/2 heterozygote or wild-type cell lines were 100- to 1000-fold less sensitive to PARP inhibitors than cells deficient in BRCA1 and 2 [36, 37]. The conclusion of both studies was that the BRCA-deficient cells were selectively sensitive to PARP inhibition by a mechanism of ‘synthetic lethality: cancer cells are selectively sensitive to the inactivation of two genes or pathways when inactivation of either gene or pathway alone is non-lethal. PARP inhibitors inhibit the repair of DNA SSBs by the BER/SSBR pathway leading to cell death [38].

If the cell cannot initiate HR, as is the case with BRCA1/2-mutant tumors, it resorts to more error-prone pathways, such as non-homologous end joining or single-strand annealing, which can cause gross chromosomal mutations, growth inhibition, and eventual cell death [39].

Then, patients with EOC with germline mutations in either *BRCA1* or *BRCA2* genes exhibit impaired ability to repair DNA DSB via HR, and show a heightened sensitivity to inhibitors of the BER pathway [40].

The identification of BRCA mutations represented a significant breakthrough in the management of breast and OC families, enabling the introduction of risk assessment and genetic counseling. Over 2000 distinct mutations and sequence variations have been identified with BRCA1 mutations more common, occurring approximately twice as frequently as BRCA2 [41].

Data from 26 observational clinical studies of 3879 women with OC reported that those with BRCA-mutated cancers have a better outcome following cytoreductive surgery and platinum based chemotherapy than their non-BRCAm counterparts [42]. The Cancer Genome Atlas Research Network carried out an analysis in which the sequencing of 316 stage-II–IV HGSOC was compared the matched normal DNA. BRCA1 and BRCA2 germline mutations were identified in 9 and 8 % of the cases, respectively. An additional 3 % showed somatic mutations of the BRCA genes; therefore, a total of 20 % of HGSOC exhibited a BRCA mutation [43].

Many sporadic EOC share pathological and clinical traits of BRCA mutation-associated cases, in the absence of a gBRCAm. This condition, in which a HR DNA repair defect is present, but no germline BRCA1 or BRCA2 mutation is detected, is termed as “BRCAness” [40, 44].

Recent reports proposed alternative models of synthetic lethality. For example, PARP inhibitors themselves can be directly toxic to cells by trapping PARP-1 and 2 and forming PARP-DNA complexes; this could lead to replication forks obstruction, which require BRCA dependent HRR to be resolved [45].

Interestingly, depleting PARP through siRNA has been shown to be less cytotoxic than depleting PARP with PARPi [46] (Fig. 1).

## Clinical trials of single parp inhibitor

### Olaparib

Olaparib is the first PARPi to be introduced in clinical practice in relapsed EOC. In the expansion phase of the phase I trial, olaparib 200 mg twice day showed a 28 % radiologic response in relapsed BRCA1/2 mutated EOC, with a response duration of 7 months [47] (Table 1).

**Table 1:** PARP inhibitors in ovarian cancer—efficacy data

Authors	Drug	Ph	Pts	Lines of therapy	BRCA status			Pl. Sens. n (%)	ORR (%)	p	mPFS (m)	p	mOS (m)	p
					mt n (%)	wt n (%)	unk n (%)							
Liu et al. [51]	OLA+CED	II	44	II–IV	23 (52)	12 (27.3)	9 (20.5)	S	79.6	0.002	17.7	0.005	nr	nr
	OLA		46		24 (52.2)	11 (23.9)	11 (23.9)	S	47.8	nr	9		nr	nr
Ledermann et al. [53]	OLA	II	136	III–V	74 (57)	57	5	S	nr	nr	8.4 all	0.0001 all	29.8 all	0.44 all
											11.2 mt	0.0001 mt	34.9 mt	0.19 mt
											7.4 wt	0.0075 wt	24.5 wt	0.96 wt
	PLA		129		62 (64)	61	7	S	nr	nr	4.8 all	nr	27.8 all	nr
											4.3 mt		31.9 mt	
											5.5 wt		26.2 wt	
Oza et al. [54]	CBDC+P+OLA	II	81	II–IV	20 (25)	nr	nr	S	64	nr	12.2 all	0.0012 all	33.8 all	0.44 all
											NR mt	0.0015 mt	NR mt	0.69 mt
	CBDC+P		81		21 (26)	nr	nr	S	58	nr	9.6 all	nr	37.6 all	nr
											9.7 mt		39.2 mt	
Coleman et al. [58]	VEL	II	50	II–IV	50 (100)	0	0	30 (60) Res	0 CR	nr	8.8	nr	19.7	nr
									20 PR					
									53 SD					
								20 (40) S	10 CR					
									25 PR					
									40 SD					
Landrum et al. [61]	PLD+CBDC+BV+VEL	I	40	II–IV	nr	nr	100	S	68	28 CR 40 PR 12 SD	nr	nr	nr	nr
Domchek et al. [56]	OLA	II	137	III, IV	137 (100)	0	0	39 S	34 All	nr	6.7 all 9.4 S 5.5 Res	nr	nr	nr
								81 Res 14 Ref	46 S					
									30 Res					
									14 Ref					
Kummar et al. [60]	OLA+CYC	II	35	II–X	16	1	43	nr	nr	nr	2.1 all	0.68	nr	nr
	CYC		37		14			nr	nr		2.3 all			
Kaye et al. [49]	OLA 200 mg	II	32	II–VI	(100)	0	0	18 (56.3) Res	25	0.31 0.11 0.13	6.5	0.78 0.63	nr	nr
								14 (43.7) S						
	OLA 400 mg		32		(100)	0	0	16 (50) Res	31		8.8			
								15 (46.9) S						
								1 (3.1) unk						
	PLD		33		(100)	0	0	14 (42.4) Res	18		7.1			
								19 (57.6) S						
Matulonis et al. [57]	OLA	I/II	273	IV–XIV	(100)	0	0	75 (27.5) S	36 all	nr	nr	nr	nr	nr
								119 (43.6) Res	48 S					
								79 (28.9) unk	28 Res					
									35 unk					
Kaufman et al. [55]	OLA	II	193	III–VI	(100)	0	0	Res	31.1	nr	7	nr	16.6	nr
Drew et al. [65]	RUC	II	45	II–VII	(100)	0	0	Res	11.1	nr	nr	nr	nr	nr
McNeish et al. [87]	RUC	II	135	II–V	35	56 LOH+ 44 LOH−	0	S	66 mt	nr	nr	nr	nr	nr
									32 wt					
									11 wt					
Shapira-Frommer et al. [66]	RUC	II	35	III–V	(100)	0	0	S	65	nr	nr	nr	nr	nr
Bell-McGuinn et al. [74]	INI	II	12	II–XV	(100)	0	0	9 (75) Res	0 CR	nr	1.6	nr	11.4	nr
								3 (25) S	0 PR					
									1 SD					
Birrer et al. [73]	INI+GC	II	19	II–VIII	nr	nr	nr	Res	31.6	nr	5.9	nr	nr	nr
Audeh et al. [48]	OLA 400 mg	II	33	I–X	(100)	0	0	13 S	33 all	nr	5.8	nr	nr	nr
								20 Res	6 CR					
									27 PR					
									36 SD					
	OLA 100 mg		24					6 S	13 all 0 CR 13 PR 29 SD		1.9			
Gelmon et al. [50]	OLA	II	63	I–X	17	46	0	20 S-wt	41 all-mt	nr	7.3 all	nr	nr	nr
								5 S-mt	0 CR-mt		7.4 mt			
								26 Res-wt	41 PR-mt		6.4 wt			
								12 Res-mt	35 SD-mt					
									24 all-wt					
									0 CR-wt					
									24 PR-wt					
									39 SD-wt					

*OLA* Olaparib, *VEL* Veliparib, *CED* Cediranib, *RUC* Rucaparib, *NIR* Niraparib, *INI* Iniparib, *PLA* Placebo, *unk* unknown, *mt* mutated, *wt* wild type, *Pts* patients, *nr* not reported, *P* Paclitaxel, *CBDC* Carboplatin, *CYC* Cyclophosphamide, *BV* Bevacizumab, *Res* platinum resistant, *Ref* platinum refractory, *LOH+* loss of heterozygosity high, *LOH−* loss of heterozygosity low, *GC* gemcitabine/carboplatin, *PLD* Pegylated liposomial doxorubicin

Monotherapy with olaparib was then investigated in a non-randomized trial, in which it was administered in two schedules: 100 mg twice daily and 400 mg twice daily. Enrolled patients were heavily pretreated EOC patients with BRCA1 and BRCA2 germline mutations. 100 mg twice daily showed a 13 % ORR, while olaparib 400 mg twice daily achieved a 33 % ORR. These results were not linked to BRCA1 or BRCA2 mutations. Similar results were seen in platinum-sensitive and in platinum-resistant EOC. The most frequent adverse events (AEs) were fatigue and nausea. Two grade 5 AEs occurred: a cardiac failure and an intestinal perforation. A patient developed a myeloid leukemia 9 months after the discontinuation of olaparib [48] (Table 2).

**Table 2:** PARP inhibitors in ovarian cancer–G3/G4 Adverse Events

	OLA (%)	OLA+CED (%)	OLA+CT (%)	VEL (%)	VEL+CHT (%)	NIR (%)	RUC (%)	INI (%)
Nausea	0–8	5	1	1	0	0	3	0
Vomiting	0–4	0	0	0	0	0.5	0	0
Diarrhoea	0–5	23	0	0	0	0	0	8.3
Dehydration	0	0	0	0	0.4	0	0	0
Hyponatremia	0	0	0	0	0.7	0	0	8.3
Abdominal pain	0–8	0	0	0	0	0	1	0
Headache	0	5	1	0	0	0	0	0
Constipation	0–2	0	0	0	0	0	0	0
Decreased appetite	0–2	0	1	0	0	0.5	0	0
Upper abdominal pain	0	0	0	0	0	0	1	0
Arthralgia	0–1	0	0	0	0	0	0	0
Back pain	0–2	0	0	0	0	0	0	0
Asthenia/fatigue	3–11	27	7	0	0	1.5	6	0
Abdominal distension	0–3	0	0	0	0	0	0	0
Leucopenia	0–2	0	5	0.5	0.7	0	0	0
Neutropenia	0–9	0	43	0.5	0.7	1.5	0	0
Lymphopenia	0–4	0	0	0	4.8	0.5	0	8.3
Thrombocytopenia	0	0	6	0.5	0.4	3.5	0	0
Anemia	0–20	0	9	0	0.7	3	0	0
Hypertension	0	41	0	0	0	0	0	8.3
Peripheral sensory neuropathy	2	0	0	0	0	0	0	0
Drug hypersensitivity	0	0	5	0	0	0	0	0
Dyspnea	4	0	0	0	0	0	0	0
Increased AST	0	0	0	0	0	0	0	8.3
Increased ALT	0	0	0	0	0	0	0	16.7
Increased activated partial thromboplastin time	0	0	0	0	0	0	0	8.3
Hyperbilirubinemia	0	0	0	0	0	0	0	8.3
Increased alkaline phosphatase	0	0	0	0	0	0	0	16.7
Increased international normalized ratio	0	0	0	0	0	0	0	16.7
Malignant pleural effusion	0	0	0	0	0	0	0	8.3
Gastro-oesophageal reflux disease	4	0	0	0	0	0	0	0
Death	0	0	0	0	0	0	0	8.3

*OLA* Olaparib, *VEL* veliparib, *CED* cediranib*, RUC* rucaparib, *NIR* niraparib, *PLA* placebo, *unk* unknown, *mt* mutated, *wt* wild type, *Pts* patients, *NR* not reported, *P* Paclitaxel, *CBDC* Carboplatin, *Res* platinum resistant, *Ref* platinum refractory, *CT* chemotherapy

In a phase II trial [49] olaparib was tested at two dose-levels, 200 and 400 mg twice daily, versus pegylated liposomal doxorubicin (PLD), given 50 mg/m^2^ every 28 days, in a population of BRCA1/2 mutated EOC patients, with a platinum-free interval of less than 12 months. Primary endpoint was investigator assessed PFS, secondary endpoints were ORR, duration of treatment response (DOR), tumor size, OS, safety and tolerability, health-related quality of life (QoL). Ninety-seven patients were enrolled: 32 to olaparib 200 mg twice daily arm; 32 to olaparib 400 mg twice daily arm and 33 in the PLD arm. No significant differences in PFS were observed between groups. Also ORRs and DOR were not statistically different. A significant difference was achieved by olaparib 400 mg vs PLD in Ca125 response (Table 1). Most common AEs in olaparib group were fatigue, anemia, gastrointestinal disorders and rash, mostly < grade 2. In the PLD arm grade 3 AEs were more frequent, in particular stomatitis, palmar-plantar erythrodysesthesia and rash. Grade 3 anemia was more frequent in the olaparib 400 mg arm than in the PLD arm. There were no significant differences in the health-related QoL between arms (Table 2).

Monotherapy with olaparib 400 mg twice daily has been investigated in another open-label non-randomized phase II trial [50] in heavily pre-treated patients, carrying BRCA1 or BRCA2 mutations, with EOC or breast cancer. Patients were divided in 4 cohorts according to primary tumor and BRCA mutational status. Overall 86 patients were evaluable for objective response: 63 in OC cohort, 23 in breast cancer cohort. ORR was greater for BRCAm then for non-mutated. The DCR in the OC population was 66 %. Five patients of 64 in OC cohort discontinued treatment due to AEs. In post hoc ORR in patients with platinum-sensitive OC were 50 % in the BRCA1 or BRCA2 negative cohort and 60 % in the BRCA1 or BRCA2 positive mutation cohort. Platinum-resistant OC responses were seen in 33 % of those in the mutation-positive cohort, but in only 4 % of those in the BRCA1 or BRCA2 negative cohort (Table 1).

Olaparib has also been investigated in association with cediranib [51], a VEGR 1-2-3 oral tyrosine-kinase inhibitor, in a phase II trial, showing 9.0 months PFS for olaparib arm and 17.7 months for cediranib and olaparib arm. The association showed to be more effective in BRCA wild type patients, while in BRCAm patients the differences were smaller (Table 1). Adverse events were more common in the association arm (Table 2).

Olaparib has been evaluated, vs placebo, as maintenance therapy in patients with recurrent platinum-sensitive EOC, fallopian tube cancer and primary peritoneal cancer. The first results showed a PFS increase for olaparib against placebo, with no statistical difference for OS [52]. Results were even better in BRCAm population. Data for OS were not mature, showing no statistically significant differences between groups (Table 1). QoL did not differs between groups [53].

Preclinical data showed that olaparib could potentiate the effect of DNA-damaging chemotherapy. This hypothesis has been investigated by Oza et al. [54], that evaluated carboplatin and paclitaxel alone or in combination with olaparib 200 mg twice daily in patients with EOC, fallopian tube cancer or primary peritoneal cancer progression-free for at least 6 months. After 4–6 cycles of combination treatment, patients in the experimental arm continued olaparib monotherapy 400 mg twice daily until disease progression or toxicity. 162 patients were randomly assigned to the two groups; BRCAm status was known for 107 patients, 41 were mutated. At the primary analysis olaparib arm showed a significant PFS increase. This increase was even bigger in BRCAm patients. OS did not significantly differed between groups, like the percentage change in tumor size, the proportion of patients with objective response, the Ca125 response and the ovarian cancer response. The exploratory analyses of time to first subsequent therapy or death and time to second subsequent therapy or death showed a significant benefit in time to first subsequent therapy or death in favor of the olaparib plus chemotherapy group, but no significant difference in time to second subsequent therapy or death between the groups. In BRCAm patients the use of olaparib led to a significant increase in these criteria (Table 1). Adverse events were more frequent in the olaparib arm, with an increased rate of alopecia, nausea, neutropenia, diarrhoea, headache, periferal neuropathy and dyspepsia, with a higher rate of grade 3 AEs (Table 2).

In another single arm phase II trial [55] olaparib has been evaluated in several kind of cancer with BRCA1/2 germline mutations. The 298 patients enrolled had EOC, breast cancer, pancreatic cancer and prostate cancer, all progressed after standard therapies. Patients were treated with olaparib 400 mg twice daily until disease progression or toxicity. Of the 298 patients enrolled, 178 had EOC, 11 had primary peritoneal cancer and 4 had fallopian tube cancer. For all patients ORR was 26.2: 31.1 % for EOC; 12.9 % for breast cancer; 21.7 % for pancreatic cancer; 50 % for prostate cancer. Median duration of response was 208 days, 225 for EOC; stable disease at 8 weeks was achieved in 41.6 % patients (40.4 % for EOC). Response were similar between BRCA1 and BRCA2 mutation carriers. Median PFS was 7, 3.7, 4.6 and 7.2 months for ovarian, breast, pancreatic and prostate cancer. Median OS was respectively 16.6, 11, 9.8 and 18.4 months. The most common Grade 3 AEs were anemia and fatigue.

About EOC population of the same trial, in particular patients treated for more than 3 lines of chemotherapy, further data were published by Domcheck et al. [56]. In addition to the previously published data, that were encouraging, we can see how the platinum sensitivity can influence the action of olaparib, indeed Platinum-sensitive population showed better outcome in all the endpoints. Serious AEs were reported in 30 % patients, frequently anemia, abdominal pain, intestinal obstruction and pleural effusion (Table 2).

A pooled analysis [57] of all EOC patients with BRCA1/2 germline mutations, treated with olaparib monotherapy 400 mg twice daily, showed that the treatment is effective and associated with durable responses. The ORR declined with the increase of the previous lines of treatment (Table 1). The 50 % of patients experienced Grade 3 AEs, with 38 % interruptions due to adverse events, the most common cause being vomiting and anemia (Table 2).

### Veliparib

Coleman et al. [58] evaluated the use of single agent veliparib in relapsed EOC in a phase II trial. Fifty-two patients with relapsed BRCA1-2 mutated EOC, were enrolled to receive Veliparib 400 mg BID until progression or toxicity. The most common hematological toxicities were anemia and leukopenia, mostly grades 1–2 (Table 2).

There were 2 complete responses (CR) and 11 partial responses (PR), and 24 SD (Table 1). 27 patients were progression-free at 6 months. Veliparib showed objective response in platinum-sensitive and in platinum-resistant populations.

Veliparib was also evaluated in association with chemotherapy. After a phase I trial [59], in which veliparib was administered in association with oral cyclophosphamide with encouraging results, Kummar et al. [60] enrolled 75 patients with BRCAm ovarian, primary peritoneal and fallopian tube cancer, progressed after at least 1 line of standard therapy, to evaluate objective response of veliparib plus cyclophosphamide versus cyclophosphamide alone. The addition of veliparib did not improve ORR on cyclophosphamide alone, for this reason patient accrual was early closed.

The use of veliparib was also evaluated in association with doublets. In a phase I trial [61] veliparib was administered in association with carboplatin, PLD and bevacizumab (BV). Objectives of the study were to determine maximum tolerated dose (MTD) and dose limiting toxicity (DLT) of this association. Patients with EOC, fallopian tube cancer and primary peritoneal cancer, progressed after 1 line of chemotherapy, with at least 6 months of platinum-free interval were eligible. The addition of veliparib to a platinum-based chemotherapy showed an important effect on bone marrow, with a great increase in thrombocytopenia and neutropenia, while BV did not add significant toxicity to this combination.

### Rucaparib

Rucaparib is a PARPi active against both PARP1 and PARP2 [62]. Furthermore it showed some activity against tankyrase TANK1, PARP5A and PARP5B [63]. In preclinical models rucaparib showed activity not only in BRCA-mutation carriers, but also in deficient-HR, like XRCC3 and epigenetically BRCA silenced [64].

In the trial by Drew et al. [65] i.v. rucaparib was administered daily for 5 days every 21 days, in patients with breast and EOC. The trial was divided in two stages: stage 1 was a short dose-escalation phase; in stage 2 efficacy and safety of rucaparib were evaluated at the dose of the stage 1. After the introduction of oral rucaparib, study design was amended and trial reopened to investigate oral rucaparib. The oral rucaparib starting dose was set at 92 mg once daily. Primary endpoints were ORR and toxicity of i.v and oral rucaparib. Secondary endpoints were determining a tolerable and effective dosing regimen for oral rucaparib, time to progression, OS. 78 patients were enrolled, 48 patients were BRCA1 m, 26 were BRCA2 m. Of the 78 patients enrolled, 51 had EOC and the median number of prior chemotherapy was 2. Rucaparib showed interesting results, in particular achieving a lot of SD. In EOC patients the greater results were seen in patients with the longest platinum free interval (Table 1).

Oral rucaparib was well tolerated up to a dose of 480 mg per day. No DLTs were seen in the i.v. phase of the study. The most common AEs were fatigue and nausea (Table 2).

Saphira-Frommer et al. [66] evaluated rucaparib in relapsed EOC patients. Patients received oral rucaparib 600 mg BID in 21 day cycles until disease progression. The primary endpoint was ORR by RECIST 1.1., RECIST/CA-125 ORR was 81 %. The most common treatment-related AEs (generally grade 1/2) were nausea, anemia, ALT/AST elevations, fatigue, and asthenia (Table 2).

### Niraparib

Niraparib, a PARP1 and PARP2 inhibitor, has been studied in a phase I dose-escalation trial [67]. In the part A of the trial, eligible patients had several kinds of malignancies, not suitable for standard treatments. In the part B of the trial only patients with sporadic platinum-resistant EOC and castration-resistant prostate cancer were enrolled. At the end of the part A, 400 mg was found to be the MTD, however 300 mg was defined to be the recommended dose in phase II trials.

Overall, 77 patients showed a response according to RECIST criteria. Among the patients in the part A, 29 showed mutations in BRCA 1 or BRCA2. 22 patients had EOC. Twenty of these 22 patients had measurable disease, showing PR in 8 patients (40 %) with doses between 80 and 400 mg. Three of 9 patients with platinum-resistant EOC showed RECIST and Ca125 responses; another patient had SD for 120 days.

### Iniparib

Iniparib was initially developed as a prodrug of the most reactive INOBA, a molecule with a powerful PARP-inhibition. Preclinical studies showed an anti-proliferative effect in triple-negative breast cancer cell lines, causing an arrest in G2/M phase; [68] however in vitro studies showed that iniparib had not a classic PARP inhibition. Also other evidences underlined this particular action of iniparib in different cell lines, in particular HR deficient cell lines exposed to veliparib, olaparib and iniparib had a sensitivity to the first two drugs, while iniparib had not an action in HR deficient cells [69]. While in triple-negative breast cancer iniparib showed interesting results in a phase II trial [70], not confirmed in the phase III [71], in EOC addition of iniparib did not reached positive results [72–74].

### Talazoparib

Talazoparib is an oral PARP-inhibitor that exhibits cytotoxic activity at a lower concentration than other PARP-inhibitors. In a phase I trial [75] recommended phase II dose was established at 1 mg/die. The most frequent dose-limiting toxicity was thrombocytopenia. Talazoparib showed interesting results in heavily pre-treated small-cell lung cancer and in BRCAm breast and EOC. Phase II trials are ongoing [76, 77].

## Resistance to parp-inhibition

Tumor heterogeneity could be a possible cause for resistance to PARP-inhibition. This phenomenon is consistent with classical Darwinian evolutionary theory [78].

Resistance to PARP-inhibition could depend on different mutations in BRCA genes: analysis of BRCA genes showed that the C- and the N-terminal domains are fundamental for the functioning of PARP-inhibition [79]. For example, the level of genomic instability of mouse tumors carrying the BRCA1-C61G RING inactivating mutation is identical to that of BRCA1-null tumors, tumor cells with BRCA1-C61G RING inactivating mutation develop a resistance to PARP-inhibition [80].

Rottenberg et al. [81] identified a possible mechanism of resistance in BRCA-deficient murine model treated with olaparib, caused by an up-regulation of Abcb1a/b genes encoding P-glycoprotein efflux pumps. The presence of this pump causes efflux of the drug outside the cell, limiting its activities. The coadministration of p-glycoprotein inhibitor tariquidar reversed this resistance.

Edwards et al. [82] showed in CAPAN1 pancreatic cell lines that resistance to PARP-inhibition can be acquired by deletion of a mutation in BRCA2. This second mutation could by-pass or correct the original mutation, restoring the physiological activity of HR.

Another possible mechanism of resistance to PARP-inhibition is loss of 53BP1. In murine model, somatic loss of 53BP1 causes the restoration of HR. In addition it seems that the same loss could be responsible for chemotherapy resistance in the same population [83].

## Conclusions

PARP inhibition leads to the persistence of spontaneously occurring SSBs and subsequent formation of DSBs, as the SSBs stall and collapse replication forks. These DSBs cannot be repaired by the defective HR pathway in BRCA-mutated cells, resulting in cell death. PARPi induce synthetic lethality in BRCA-deficient tissues. BRCA1/2-deficient cancers are now recognized as the target of a class of drugs known as PARPi. Deficiency of either PARP or BRCA alone has no impact, but deficiency in both leads to a lethal effect.

These data suggest that there is likely to be a role for PARPi in the treatment of EOC. PFS appears to be improved in women with recurrent platinum-sensitive disease, with manageable side effects. However, beneficial effects in terms of OS have not been adequately demonstrated and more data are required to determine whether longer PFS translates into an improved OS. More data are expected from ongoing phase III clinical trials. The European Medicines Agency (EMA) approved olaparib for monotherapy for the maintenance treatment of adult patients with platinum sensitive relapsed BRCAm (germline and/or somatic) HGSOC, fallopian tube, or primary peritoneal cancer who are in response to platinum-based chemotherapy in 2014.

In the absence of more refined understanding of PARPi action, *BRCA1*/*2* mutation status has been the most extensively studied predictor of PARPi sensitivity to date. When PARPi are administered as single agents in the relapsed setting, *BRCA1*/*2*-mutated OC has a 30–45 % ORR, with better responses in platinum-sensitive EOC.

In the meantime, two lines of clinical development were actively pursued. First of all, the concept that PARP inhibition in EOC might have utility extending beyond those cases associated with BRCAm. The key property predicting efficacy is HRD, and in 2011, Levine’s work within the Cancer Genomic Atlas framework indicated that up to 50 % of cases of HGSOC might be candidates for PARPi, based on a range of genetic defects in addition to BRCA 1 or 2 germline and somatic mutations [43].

The clinical relevance of the observations was assessed in a clinical trial published in 2011, which demonstrated efficacy of olaparib in a series of patients with sporadic, BRCA wild-type EOC, albeit at a slightly lower level (24 %) and confined mainly to patients with platinum-sensitive disease [48, 49].

The second line of investigation, which led directly to the approval of olaparib by regulatory authorities in Europe, examined the use of the drug as a form of maintenance therapy. The key randomized trial involved patients with platinum-sensitive relapsed disease who received single-agent olaparib or placebo following platinum-based treatment. The trial had not selected for patients with BRCAm, and mutation status was initially unknown in the majority of cases (64 %) [52].

However, retrospective analysis indicated that 136 patients (51 %) were positive for BRCA 1 or 2, and the treatment benefit in this subgroup was even more marked (HR = 0.17).

Other notable features in this retrospective analysis included the positive benefit in patients with BRCA wild-type disease and in those with sBRCAm and both these observations will be taken forward in subsequent trials involving olaparib as well as two other PARPi (niraparib and rucaparib).

Accumulating evidence suggests that PARPi may have a wider application in the treatment of sporadic EOC. Up to 50 % of HGSOC patients may exhibit HRD through mechanisms including gBRCAm, sBRCAm, and BRCA promoter methylation [84].

It is now well established that gBRCAm EOC have a relatively distinct clinical behavior characterised by an earlier age at diagnosis, improved survival, visceral distribution of disease, higher response rates to platinum and certain non-platinum chemotherapy agents and sensitivity to PARPi [85–87].

However, it became increasingly apparent that a proportion of sporadic EOC also share pathological and clinical traits of BRCA mutation-associated cases, but in the absence of a gBRCAm. This concept, termed as ‘BRCAness’ over a decade ago, now describes the situation whereby a HR DNA repair defect is present, but no germline BRCA1 or BRCA2 mutation is detected [44].

This is becoming increasingly relevant clinically as the activity of PARPi has now been demonstrated in trials to extend beyond gBRCAm EOC [87].

The first demonstration of clinically meaningful activity of a PARPi in EOC patients without a gBRCAm was provided by Gelmon et al. [50] in a phase II study which included patients with HGS/undifferentiated OC with unknown BRCA status or BRCA-negative treated with olaparib.

Evidence for attributing the ‘BRCAness’ phenotype to HRD due to the mechanisms other than germline mutations in BRCA1 or BRCA2 comes from several studies [88].

HRD leads to massive genomic instability resulting in tumour formation. In addition, HRD cancers are potentially sensitive to drugs that induce lesions that are normally repaired by the HR pathway. Furthermore, the synthetic lethal interaction described with PARPi may be exploited beyond gBRCAm EOC in the context of HRD. Several genetic lesions causing HRD include germline and somatic BRCAm as well as mutations of genes such as ATM, CHEK2, BARD1, BRIP1, MRE11, RAD50, NBS1, RAD51C, RAD51D and PALB2. It is clear that if the indication for PARPi is to expand into a BRCA-wild type population, robust tests with a high probability of determining HRD status are needed [89–92].

In contrast, not all patients with deleterious *BRCA1* or *BRCA2* mutations at diagnosis respond to PARPi. In cell lines, secondary somatic mutations in *BRCA1*- or *BRCA2*-mutant cancer cells can restore protein expression, reconstitute HR, and confer resistance to PARPi and platinum.

Secondary mutations that restore BRCA1 and BRCA2 also predict platinum and PARPi resistance in the clinical setting. It seems that approximately 45 % of recurrent platinum-resistant *BRCA1*/*2*-mutated EOCs have secondary somatic mutations.

Interestingly, clinical cancer specimens most commonly sustain secondary somatic mutations that revert the mutant allele to wild-type sequence, making secondary mutations highly predictive of response but technically difficult to identify.

In addition to reversion mutations, HR can be restored in other ways. Some mutant *BRCA1* alleles encode proteins that are potentially functional but degraded rapidly. Stabilization of these mutant proteins can restore HR and confer PARPi resistance without any secondary *BRCA1* mutation.

Likewise, decreased expression of 53BP1, which ordinarily channels DSB repair to NHEJ, restores HR and confers PARPi resistance in *BRCA1*-mutant cells despite the continued absence of BRCA1 protein.

The extent to which these mechanisms contribute to PARPi resistance in clinical EOC remains to be fully defined. Despite the current focus on *BRCA1*/*2* mutation carriers with EOC, responses are not limited to this group. EOCs with somatic *BRCA1*/*2* mutations seem to be as likely to benefit from PARPi maintenance therapy as those with inherited mutations, although the number of treated patients with somatic mutations is small. Moreover, germline or somatic mutations in other genes critical to HR correlate with platinum sensitivity in EOC and might also predict PARPi response.

In addition to mutations, other processes, including epigenetic alterations and changes in expression of microRNAs or transcription factors, could in principle impair HR and confer PARPi sensitivity. *BRCA1*promoter hypermethylation, which downregulates BRCA1 expression, occurs in 10–15 % of OCs and has been proposed as a mechanism of HRD.

However, data from The Cancer Genome Atlas and others fail to correlate *BRCA1* hypermethylation with increased platinum sensitivity or improved survival, suggesting that epigenetic BRCA1 downregulation may have a less profound impact on HR and PARPi sensitivity than inactivating *BRCA1* mutations. In short, improved understanding of PARP biology and HRD is providing important new clues for predicting PARPi responders vs non responders.

Questions remain about how best to use PARPi, whether to use in combination with chemotherapy or as maintenance alone. Possible combination treatment with PARPi include anti-angiogenic agents or in combination with cyclophosphamide or weekly paclitaxel. Pre-clinical data suggest that inhibiting vascular endothelial growth factor receptor (VEGFR) may lead to down-regulation of DNA-repair activity by DNA-repair proteins, ERCC1 and XRCC1 [93]. The mechanism of this therapeutic approach is to induce HRD in otherwise HRR-competent cancers by altering the tumour microenvironment through hypoxia, or to combine PARPi with agents that can downregulate HRR, such as vascular endothelial growth factor (VEGF) inhibitors. This may lead to increased DNA damage and, thereby, increase susceptibility to the effects of PARPi. This concept, known as ‘contextual’ synthetic lethality, could further broaden the application of this class of drugs and is the rationale behind many ongoing clinical trials.

Clinical studies of PARPi in combination with chemotherapy agents are ongoing (Table 3). Future studies should include OS and QoL as important outcomes. In women with platinum-resistant EOC objective responses to both PARPi and PLD were demonstrated at higher levels than previous studies of women with platinum-resistant EOC in non-selected populations [49].

**Table 3:** PARP inhibitors in ovarian cancer—phase II–III ongoing studies

ClinicalTrials.gov Identifier	Responsible party	Ph	Drugs
NCT01033292	Sanofi	II	CBDC/GEM with INI in Pts with platinum-resistant recurrent EOC
NCT01033123	Sanofi	II	CBDC/GEM with INI in Pts with platinum-sensitive recurrent EOC
NCT00677079	Sanofi	II	INI in Pts with BRCA-1 or BRCA-2 associated advanced EOC
NCT01482715	Clovis Oncology, Inc.	II	RUC in Pts with gBRCA Mutation EOC
NCT01891344	Clovis Oncology, Inc.	II	RUC in Pts with platinum-sensitive, relapsed, HGSOC (ARIEL2)
NCT01968213	Clovis Oncology, Inc.	III	RUC as switch maintenance after platinum in relapsed HGSOC (ARIEL3)
NCT00664781	Cancer Research UK	II	RUC in known carriers of a BRCA 1 or BRCA 2 mutation with advanced EOC
NCT01690598	Vejle Hospital	II	VEL and TOP for Pts with platinum-resistant or partially platinum-sensitive relapse of EOC with negative or unknown BRCA status
NCT01472783	Vejle Hospital	II	VEL for Pts with BRCA germline mutation and platinum-resistant or partially platinum-sensitive relapse of EOC
NCT01113957	AbbVie (Abbott)	II	VEL with TEM vs PLD alone in subjects with recurrent HGSOC
NCT01306032	National Cancer Institute	II	VEL in combination with metronomic oral CYC in refractory BRCA-positive EOC
NCT02470585	AbbVie	III	CBDC/P with or without concurrent and continuation maintenance VEL in subjects with previously untreated stages III or IV HGSOC
NCT01540565	National Cancer Institute	II	VEL in persistent or recurrent EOC with germline BRCA1/BRCA2 mutation
NCT02392676	AstraZeneca	III	PLA controlled study of OLA maintenance in Pts With platinum sensitive relapsed EOC and loss of function somatic BRCA mutation(s) or loss of function mutation(s) in tumour homologous recombination repair-associated genes
NCT01874353	AstraZeneca	III	PLA controlled study of OLA maintenance in platinum sensitive relapsed BRCA mutated EOC Pts with a complete or partial response following platinum based CT
NCT02571725	New Mexico Cancer Care Alliance	II	Combination of OLA and TREM, in BRCA1 and BRCA2 mutation carriers with recurrent EOC
NCT01844986	AstraZeneca	III	OLA maintenance in Pts With BRCA mutated advanced EOC following first line platinum based CT
NCT01081951	AstraZeneca	III	OLA With P and CBDC vs P and CBDC alone in Pts with platinum sensitive advanced EOC
NCT02503436	AstraZeneca	II	OLA treated BRCAm EOC POPULATION
NCT02282020	AstraZeneca	III	OLA vs Physician’s choice single agent CT for platinum sensitive relapsed EOC in Pts carrying germline BRCA1/2 mutations
NCT02484404	National Institutes of Health Clinical Center (National Cancer Institute)	II	Anti-programmed death ligand-1 antibody MEDI4736 in combination With OLA or CED for advanced solid tumors and advanced or recurrent EOC
NCT02340611	University Health Network, Toronto	II	Combination CED-OLA at the time of disease progression on OLA in EOC
NCT01661868	Ursula A. Matulonis, MD, Dana-Farber Cancer Institute	II	OLA for Pts with recurrent BRCA deficient EOC with no prior PARP exposure or prior PARP inhibitor exposure
NCT02477644	ARCAGY/GINECO GROUP	III	OLA or PLA in with platinum-taxane and BV and as maintenance therapy
NCT02345265	National Cancer Institute	II	OLA and CED for the treatment of recurrent EOC
NCT02208375	M.D. Anderson Cancer Center	II	OLA With AZD2014 or AZD5363 for recurrent endometrial, triple negative breast, and ovarian, primary peritoneal, or fallopian tube cancer
NCT02502266	National Cancer Institute	II/III	CED and OLA compared to CED or OLA alone, or standard of care CT in women with recurrent platinum-resistant or -refractory EOC
NCT02489006	University Health Network, Toronto	II	OLA in Pts with platinum sensitive recurrent HGSOC
NCT00628251	AstraZeneca	II	OLA vs intravenous liposomal doxorubicin given monthly in Pts with advanced BRCA1- or BRCA2-associated EOC who have failed previous platinum-based CT
NCT02485990	Sidney Kimmel Comprehensive Cancer Center	II	TREM alone or combined with OLA for recurrent or persistent EOC
NCT01116648	National Cancer Institute	II	CED and OLA for recurrent papillary-serous ovarian, fallopian tube, or peritoneal cancer or for treatment of recurrent TNBC
NCT01078662	AstraZeneca	II	OLA in Pts with advanced cancers BRCA 1 and/or BRCA2 mutation
NCT00494442	AstraZeneca	II	OLA twice daily in Pts with advanced BRCA1 or BRCA2 associated EOC
NCT00679783	AstraZeneca	II	OLA in Pts with known BRCA or recurrent HGSOC and in known BRCA or TNBC
NCT00753545	AstraZeneca	II	OLA Pts with platinum sensitive relapsed serous EOC following treatment with two or more platinum containing regimens
NCT02326844	National Institutes of Health Clinical Center (National Cancer Institute)	II	TAL in Pts with deleterious BRCA1/2 mutation-associated EOC who have had prior PARP inhibitor treatment
NCT01989546	National Cancer Institute	II	TAL in Pts with advanced solid tumors and deleterious BRCA mutations
NCT01847274	Tesaro, Inc.	III	Maintenance with NIR vs PLA in Pts with platinum sensitive EOC
NCT02354131	Nordic Society for Gynaecologic Oncology	II	NIR and/or NIR-BV combination against BV alone in HRD platinum-sensitive EOC
NCT02354586	Tesaro, Inc.	II	NIR in women with advanced, relapsed, HGSOC, fallopian tube, or primary peritoneal cancer who have received at least three previous CT regimens
NCT02657889	Tesaro, Inc.	II	NIR With PEM in Pts with advanced TNBC and in Pts with recurrent EOC
NCT02655016	Tesaro, Inc.	III	NIR maintenance in Pts With HRD-positive advanced EOC following response on front-line platinum-based CT
NCT02446600	National Cancer Institute	III	Comparing OLA or CED and OLA to standard platinum-based CT in women with recurrent platinum-sensitive EOC
NCT01434316	National Cancer Institute	I	VEL and DIN given together with or without CBDC
CRUK/13/023	Cancer Research UK	III	OLA with CED vs CED and PLA as maintenance therapy following platinum-based CT with CED

Reference: https://www.clinicaltrials.gov; http://www.cancerresearchuk.org

*EOC* epithelial ovarian cancer, *OLA* olaparib, *VEL* veliparib, *CED* cediranib, *RUC* rucaparib, *DIN* dinaciclib, *NIR* niraparib, *TAL* talazoparib, *GEM* gemcitabine, *TOP* topotecan, *PEM* pembrolizumab, *TREM* tremelimumab, *CYC* cyclophosphamide, *TEM* temozolomide, *PLD* pegylated liposomal doxorubicin, *PLA* placebo, *mt* mutated, *wt* wild type, *Pts* patients, *P* paclitaxel, *CBDC* carboplatin, *TNBC* triple-negative breast cancer, *Ph* phase

Beyond olaparib, other PARPi that have been tested or are currently being tested in clinical trials for EOC include veliparib, niraparib, rucaparib, talazoparib and iniparib.

Radiotherapy, inducing DNA damage by multiple mechanisms including base damage and SSB and DSB DNA, could be a fascinating partner for PARPi therapy [94–96]. A work by Anthony Chalmers’ group has shown that this radio-potentiation is enhanced in rapidly proliferating cells and cells defective in DNA DSB repair compared with normal tissue [97]. These data support a role for combining radiotherapy and PARPi in patients with cancer, and clinical trials are finally underway with results eagerly awaited.

Based on a body of evidence and the clinical success of immunotherapy in many malignancies, it is confirmed that blocking the programmed death 1 (PD-1) and its ligands in EOC is feasible and valid both in animal models and patients. Immunotherapy may play a significant role in the future clinical management and improve the prognosis of EOC. The phase 2 trial that first to explore the effects of nivolumab (anti-PD-1 mAb) against platinum-resistant EOC has published the safety and antitumor activity results [98].

PD-1 immune checkpoint blockade provides significant clinical benefits for melanoma patients; moreover, high mutational loads are associated with improved survival in melanoma patients but are not predictive of response to anti-PD-1 therapy, suggesting that other genomic and non-genomic features also contribute to response patterns on PD-1 checkpoint blockade therapy. Hugo et al. analyzed the somatic mutanomes and transcriptomes of pretreatment melanoma biopsies to identify factors that may influence innate sensitivity or resistance to anti-PD-1 therapy; and they find that overall high mutational loads associate with improved survival, and tumors from responding patients are enriched for mutations in the DNA repair gene BRCA2. Thus, BRCA2 loss-of-function mutations, which are expected to produce defects in HR and DSB DNA break repair, may produce specific mutational signatures or unknown effects (e.g., induction of cell death) that contribute to anti-PD-1 responsiveness [99, 100].

Moreover, considering PARPi mechanism of action, their use could further increase the mutational loads in BRCAm EOC patients; therefore it would be very interesting to evaluate the effectiveness of the combination of PARPi and anti-PD1 or anti-CTLA4 in BRCAm EOC patients, as it is ongoing in some trial (Table 3).

Furthermore, Higuchi et al. [101] used an immunocompetent BRCA1-deficient murine EOC model to compare treatment with Cytotoxic T Lymphocyte-associated Antigen-4 (CTLA-4) or PD-1/PD-L1 antibodies alone or combined with targeted cytotoxic therapy using a PARPi. Correlative studies were performed in vitro using human BRCA1(−) cells. They found that CTLA-4 antibody, but not PD-1/PD-L1 blockade, synergized therapeutically with the PARPi, resulting in immune-mediated tumor clearance and long-term survival in a majority of animals (P < 0.0001). These results support clinical testing of this regimen to improve outcomes for women with BRCAm EOC.

Actually, there are many trials in progress to address the additional populations that may have deficiencies in the HR pathway that will benefit from PARPi (Table 3). Additionally, combination trials with chemotherapy, radiation and TKIs are expanding the exploration of usage. Suggested by the cediranib and olaparib combination, combining PARPi with another agent may not require additional DNA impairment for efficacy. Trials are also underway investigating agents that impair the DNA damage repair pathway, like veliparib and dinaciclib creating synthetic lethality without additional patient selection [102] (Table 3).

Therefore, the approval of olaparib in the maintenance setting in Europe and metastatic setting in the US for patients with deleterious BRCAm in EOC is just the tip of the iceberg for the utilization for this class of agents.

## Abbreviations

EOC: epithelial ovarian cancer
PARP: Poly(ADP-ribose) polymerase
PARPi: PARP inhibitors
HR: homologous recombination
HRD: homologous recombination deficiency
BRCA: breast related cancer antigens
gBRCAm: germline breast related cancer antigens mutation
sBRCAm: somatic breast related cancer antigens mutation
HGSOC: high-grade serous ovarian cancer
SD: stable disease
RR: response rate
BV: bevacizumab
PFS: progression free survival
OS: overall survival
CSCs: cancer stem cells
SSB: single strand breaks
DSB: double strand breaks
BER: base excision repair
NER: nucleotide excision repair
MMR: mismatch repair
NAD: nicotinamide-adenine-dinucleotide
PARG: PAR hydrolysis by poly-(ADP-ribose) glucohydrolase
LigIII: DNA ligase III
polβ: DNA polymerase beta
XRCC1: X-ray cross complementing gene 1
NHEJ: non-homologous end-joining
PLD: pegylated liposomal doxorubicin
QoL: quality of life
DOR: duration of treatment response
AEs: adverse events
CR: complete responses
PR: partial responses
MTD: maximum tolerated dose
DLT: dose limiting toxicity
EMA: European Medicines Agency
VEGFR: vascular endothelial growth factor receptor
VEGF: vascular endothelial growth factor
PD-1: programmed death 1
